# Biombalance™, an Oligomeric Procyanidins-Enriched Grape Seed Extract, Prevents Inflammation and Microbiota Dysbiosis in a Mice Colitis Model

**DOI:** 10.3390/antiox14030305

**Published:** 2025-03-01

**Authors:** Mohamed Mokrani, Naima Saad, Ludivine Nardy, Elodie Sifré, Julie Despres, Amandine Brochot, Christine Varon, Maria C. Urdaci

**Affiliations:** 1 University Bordeaux, CNRS, Bordeaux INP, CBMN, UMR 5248, F-33600 Pessac, France; mohamed.mokrani@agro-bordeaux.fr (M.M.); naima.saad@unilim.fr (N.S.); ludivine.nardy@agro-bordeaux.fr (L.N.); 2 Bordeaux Sciences Agro, F-33175 Gradignan, France; 3 Groupe Berkem, 20 Rue Jean Duvert, F-33290 Blanquefort, France; julie.despres@berkem.com (J.D.); amandine.brochot@berkem.com (A.B.); 4 Univ. Limoges, LABCiS, UR 22722, F-87000 Limoges, France; 5 INSERM U1312 BRIC Bordeaux Institute of Oncology, Université de Bordeaux, F-33077 Bordeaux, France; elodie.sifre@u-bordeaux.fr (E.S.); christine.varon@u-bordeaux.fr (C.V.)

**Keywords:** OPC, grape seed extract, antioxidant, anti-inflammatory, microbiota, DSS, prebiotic

## Abstract

Inflammatory bowel disease (IBD) results from genetic factors, environmental factors, and intestinal microbiota interactions. This study investigated the effects of Biombalance™ (BB) in dextran sulphate sodium (DSS)-induced colitis in mice. BB extract exhibits high antioxidant activity, as determined by DPPH and ORAC tests. Mice were fed a standard diet, and BB was administered by gavage for ten days, before administration of 2.75% DSS in drinking water. BB significantly protected mice against DSS effects, as assessed by colon length, disease activity index (DAI) scores and colonic pathological damage. In addition, BB inhibited the expression of proinflammatory markers, such as IL-6, IL-17, CXCL1 and TNF-α, and the inflammatory mediators iNOS, TGF-β, FoxP3 and F4/80, while increasing IL-10 expression in the colon. BB modified microbiota composition, attenuating the microbial diversity lost due to DSS, increasing beneficial bacteria like *Muribaculum*, *Lactobacillus*, *Muscispirillum*, *Roseburia* and *Bifidobacterium*, and decreasing potentially harmful bacteria such as *Proteobacteria* and *Enterococcus*. Interestingly, microbiota-predicted functions using PICRUSt revealed that BB extract increases the antioxidant superpathway of ubiquinol biosynthesis, including ubiquinol-7, 8, 9 and 10 (CoenzymesQ). These findings suggest that Biombalance™ administration may help to reduce gut inflammation and oxidation, at least partly through modifications of the microbiota and its metabolites.

## 1. Introduction

Inflammatory Bowel Diseases (IBDs), including ulcerative colitis and Crohn’s disease, are multifactorial diseases closely linked to gut microbiota homeostasis [1]. The worldwide prevalence of IBD have been increased globally in the 21st century [2]. IBD symptoms encompass diarrhea, abdominal pain, fatigue, nausea, weight loss, bloody stools and fecal urgency. In addition, low-grade inflammation at the intestinal mucosa level exists, inducing modifications in natural GI functions [3].

Polyphenols are secondary metabolites of plants that actively participate in their survival by allowing plants to fight against environmental stresses, like UV radiation and pathogenic attacks, thanks to their antimicrobial and antioxidant properties [4,5]. These metabolites are biosynthetically derived from the shikimate-derived phenylpropanoid and/or polyketide pathways, harbouring more than one phenolic moiety, and lacking nitrogen-based functional groups in their basic structure [6,7]. They can be monomeric, among which are flavonoids (flavones, flavonols, flavanols, flavanones, isoflavones, anthocyanidins) and non-flavonoids (such as stilbenes and lignans), or they can be polymeric (tannins) [8]. Procyanidins, also called proanthocyanidins, are condensed tannins resulting from the condensation of flavanols, leading to oligomeric proanthocyanidins (OPCs), dimers, trimers and more highly polymerized forms [9]. Their absorption and biological activity are dependent on structural variabilities.

Grape seed extract is a rich source of procyanidins, mainly composed of gallic acid, monomeric catechin, epicatechin, epicatechin-3-O-gallate and oligomeric proanthocyanidins [10,11]. OPCs are potent free radical scavengers, much more powerful antioxidants than vitamins E, C and beta-carotene, contributing to the beneficial effects against various human pathologies, including inflammation, cancer, and cardiovascular and neurological diseases [12,13,14]. OPC-positive effects on gut health, such as intestinal barrier protection, modulation of gut hormones, immunomodulation and regulation of gut microbiota, have also been reported [15,16,17]. More specifically, OPCs have anti-inflammatory effects in the intestine by inhibiting the formation of proinflammatory cytokines [18,19,20]. Moreover, OPCs can promote beneficial bacteria, exerting prebiotic effects [6,21,22].

The positive effects of polyphenol-enriched diets on mice colitis have been reported, notably for their preventive potential, even after short-term administration [23,24]. Because OPCs can exert various health-beneficial effects, including strong anti-inflammatory properties, their use can be considered a possible adjuvant for IBD in alleviating gut inflammation and dysbiosis [19]. However, OPCs’ effects can depend on their source, type and extent of polymerization, which is essential for their absorption. Procyanidin monomers and dimers are absorbed in the small intestine relatively well, and trimers and tetramers are less well absorbed, while longer oligomers are absorbed very little. Non-absorbed forms can reach the colon and be catabolized by the microbiota into a series of low-molecular-weight phenolic acids, such as phenyl valerolactone and phenylacetic and phenylpropionic acids [25,26]. In addition, OPCs can modulate the intestinal microbiota thanks to their dual effect on bacteria: antibacterial, or prebiotic by increasing the number of positive bacterial genera in the gut, such as *Lactobacillus*, *Bifidobacterium*, *Akkermansia*, *Roseburia* and *Faecalibacterium*, as retrieved in various clinical trials [27].

Grape seed composition and extracts largely depend on the grape cultivar and the extractive method [28]. In this study, we investigate the protective effect of Biombalance™, a grape seed extract rich in proanthocyanidins from three cultivars (France), in a DSS-treated mice model of colitis, by studying microbiota and intestinal homeostasis.

## 2. Materials and Methods

### 2.1. Composition Analysis of OPC-Rich Polyphenolic Extract

Biombalance™ was extracted from three specific “champagne” grape varieties: Chardonnay, Pinot Noir and Meunier (Champagne, France).

The extract characterization and quantification of major phenolic compounds were realized by UHPLC-DAD-MS, using a UHPLC from Agilent Technologies (Santa Clara, CA, USA) and the Mass Spectrometer Esquire 6000 (Bruker Daltonics, Bremen, Germany), equipped with an electrospray ionization source. A mass of 30 mg of extract was dissolved in 6 mL of methanol/water/acetic acid (99.5/99.5/1; *v*/*v/v*). The solution was centrifuged and then injected (1 μL) onto an Agilent Zorbax SB-C18 column (2.1 mm × 100 mm, 1.8 μm), at a temperature maintained at 25 °C. The solvents used were A = water/formic acid (99.9/0.1; *v*/*v*) and B = acetonitrile/formic acid (99.9/0.1; *v*/*v*), and the flow rate was 0.4 mL/min.

The separation of procyanidins according to their degree of polymerisation was realized by HPLC-fluorescence, using a column Phenomenex LUNA HILLIC (250 mm × 4.6 mm, 5 μm) at 25 °C and a 1260 infinity fluorescence detector. The solvents used were A = acetonitrile/acetic acid (98/2; *v*/*v*) and B = methanol/water/acetic acid (95/3/2; *v*/*v*/*v*), and the flow rate was 0.8 mL/min.

The compounds were quantified by epicatechin equivalent. For oligomers, a correction factor was applied, taking into account their different fluorescence response factors [29].

### 2.2. Measure of Biombalance™ Antioxidant Activity

Two methods were used. (i) The free radical scavenging capacity (RSC) of BB extract was evaluated using the DPPH radical assay (n = 4), according to Brand-Williams et al. [30]. DPPH assays are generally classified by a sequential mechanism of electron transfer with proton loss, in which the DPPH radical accepts an electron, followed by proton transfer from antioxidant compounds. (ii) Following the manufacturer’s guidelines, the oxygen radical absorbance capacity (ORAC) was assessed using the BQC ORAC Assay Kit from Bioquochem S.L, Oviedo, Spain, in triplicate. This kit prevents the oxidation of fluorescein by peroxyl radicals in the presence of antioxidants. A free radical initiator generates the peroxyl radicals (ROO) and AAPH (2,2′-azobis-2-methylpropanimidamide dihydrochloride), gradually diminishing the fluorescence of fluorescein. Antioxidants help to protect fluorescein from free radical damage, thereby maintaining its fluorescent signal. The results are expressed as µM Trolox equivalent per gram dry weight.

### 2.3. Experiment Design

Animal experiments were carried out according to the rural and maritime fishing codes R.214-87 to R.214-126. The Experimental Animal Ethics Committee of Bordeaux University, APAFIS#17304-201810191064539, 2019 to 2024, approved the experimental protocol.

Male Balb/c SFP-grade mice at 6 weeks of age were purchased from JANVIER LABS (Lorient, France). The mice were housed under standard laboratory conditions: 22 °C, 60% humidity and a 12 h light/dark schedule. Environmental enrichment items, such as gnawing sticks and climbing structures, were provided to promote physical and mental stimulation. The animals had free access to water and food. The animals were fed a standard diet (SD (A04 diet composition in Figure S1). Throughout the experiment, training personnel performed daily health checks to monitor for signs of distress or illness. Handling was conducted gently, using refined methods. Pain management protocols were in place for any potentially painful procedures. After a week of acclimatization, the mice were randomly assigned to four groups, each containing eight mice. The BB and BB-DSS groups received 4.8 mg/day of the BB extract by gavage in 100 µL of PBS. In contrast, throughout the experiment, the control (Ctl) and DSS groups only received 100 µL of PBS. After ten days of gavage, colitis was induced by administering 2.75% (*w*/*v*) of dextran sulphate sodium (DSS) (MP Biomedicals colitis grade, 36,000–50,000, LLC, Illkirch-Graffenstaden, France) in the drinking water for seven consecutive days. Fresh DSS water was replenished daily, followed by two days of providing only water. The mice were euthanized on day 10 by cervical dislocation. The Ctl and BB groups received only drinking water.

The mice were weighed and visually observed daily, and the disease activity index (DAI) score, ranging from 0 to 4, was calculated daily. The DAI was used to assess colitis grade using body weight loss, stool consistency and rectal bleeding [31]. Feces were taken aseptically the day before the mice were sacrificed.

### 2.4. Clinical Scoring of Murine Colitis

During the DSS treatment, the DAI score was used to assess the progression of colitis. The DAI is a widely used metric for evaluating the severity of inflammation in mice with colitis. It is based on three factors: body weight loss, stool consistency and the presence of blood in the rectum [32]. Body weight loss was scored as follows: (0) no weight loss compared to initial weight, (1) weight loss of 1–5%, (2) weight loss of 5–10%, (3) weight loss of 10–20% and (4) weight loss greater than 20%. Stool consistency was assessed with (0) normal stool (solid pellet), (1) soft but pellet-shaped stool, (2) loose stool with some solidity, (3) loose stool with signs of a liquid consistency and (4) watery diarrhea. Rectal bleeding was scored as (0) no sign of blood, (1) no bleeding, (2) slight bleeding, (3) bloody diarrhea and (4) gross bleeding [33]. Moreover, colon shorting, a good indicator of inflammation in the DSS model, was measured [34].

### 2.5. Histological Evaluation of Colitis

Distal colonic tissue samples were collected and immediately fixed with 4% formaldehyde for 24 h, then washed and conserved in 70% ethanol at 4 °C. Tissue sections with a thickness of 3 μm from paraffin-embedded tissues (PETs) were processed for Hematoxylin/Eosin staining (CARLO ERBA reagent, RAL DIAGNOSTICS, Martillac, France), following previously described standard procedures [35]. The colonic epithelial lesions were evaluated microscopically and scored double-blindly using a numerical scale of 0 to 3 or 4 for each criterion, adapted from [36,37], as follows: Inflammation severity: 0, no inflammation; 1, minimal (little multifocal accumulation); 2, moderate (little multifocal coalescing inflammation or considerable multifocal non-coalescing inflammation); 3: severe (extensive multifocal and coalescing inflammation). Inflammation extension (vertical): 0, no inflammation; 1, mucosa; 2, mucosa and submucosa; 3, transmural. Hyperplasia: 0, normal; 1, <2-fold crypt height; 2, 2-fold crypt height; 3, 3-fold crypt height; 4: ≥4-fold crypt height, +/− adenomatous polyps. Crypt damage (ulceration) severity: 0, no crypt damage; 1, 1/3 of crypt damaged; 2, 2/3 of crypt damaged; 3, crypts lost with surface epithelium intact; 4, crypts lost with surface epithelium lost. Crypt damage extension (longitudinal), percentage of area involved: 0, 0%; 1, 1–25%; 2, 26–50%; 3, 51–75%; 4, >76%.

The total histological score for each sample was determined by adding the scores of inflammation severity, inflammation extension, hyperplasia and crypt damage severity, and multiplying by the score of extension (area involvement). The minimum possible score was 0, and the maximum was 56, or 0-40 if the score of crypt damage severity was not added. (Histological scoring detailed in Table S1).

### 2.6. Tissue Sampling, RNA Extraction and Real-Time PCR (RT-PCR)

Ileum, proximal and distal colon and liver samples were collected and preserved in RNAlater (Qiagen, Hilden, Germany) at 4 °C for 24 h. They were then stored at −20 °C under RNA extraction. Total RNA was isolated using the RNeasy Mini Kit (QIAGEN, Germany) supplemented with DNase treatment (DNase, Thermo Scientific, Waltham, MA, USA), according to the manufacturer’s instructions, and was quantified using Nano Drop One (Thermo Fisher Scientific, USA). A quantity of 250 ng of total RNA was reverse transcribed using a Hexamers kit (SuperScript IV, Thermo Scientific, USA). qPCR analyses were performed using CFX96 (Bio-Rad, San Francisco, CA, USA). The reaction mixture comprised SsoAdvanced™ Universal SYBR Green Supermix (Biorad, USA). Target gene copy numbers were normalized against the housekeeping gene hypoxanthine phosphor ribosyl transferase (HPRT). The primers and the PCR cycle are described in Table S2. Data were analyzed by the 2^−ΔΔCT^ method and expressed as relative abundance.

### 2.7. Stool Sampling, Fecal DNA Extraction and 16S rDNA Sequencing

One day before the end of the protocol, stools were collected in sterile tubes and stored at −80 °C. Around 100 mg of feces were accurately weighted and homogenized in Tris-EDTA buffer (Tris 0.1 mM, pH 8; EDTA 1 M (Sigma); 1 mL buffer for 200 mg of feces). Lysozyme 1:100, 300 mg/mL (Sigma, Germany) was added to the mixture and incubated at 37 °C for one hour. Then, 300 µL of the mixture was used for DNA isolation with the NucleoSpin^®^ Soil kit (Macherey-Nagel, Düren, Germany), according to the manufacturer’s instructions [38]. DNA concentration and purity were determined by Nano Drop One (Thermo Fisher Scientific, USA). For Illumina microbiota sequencing, the 16S rRNA gene’s V3–V4 region was amplified using forward primer 338F (5′-ACTCCTACGGGAGGCAGCA-3′) and reverse primer 806R (5′-GGACTACHVGGGTWTCTAAT-3′), to prepare Illumina libraries. The PCR mixture, containing 10 ng of DNA, 10 μM of each primer and PCR-grade water in a final volume of 50 μL, was prepared using the MP Taq DNA Polymerase kit (Q-Bio DNA polymerase, MP Biomedicals, Illkirch-Graffenstaden, France). PCR cycling involved an initial denaturation step at 95 °C for 5 min, followed by 30 cycles of 30 s at 95 °C, 30 s of annealing at 52 °C and 45 s of extension at 72 °C, concluding with a final extension step at 72 °C for 2 min. Subsequently, DNA amplicons were sequenced on the Illumina MiSeq platform (Genotoul, France).

The obtained sequences underwent quality assessment using GALAXY FROGS 4.1 [39] to discard low-quality sequences. Paired-end joined sequences were grouped into operational taxonomic units (OTUs), and then clustered using Swarm (aggregation parameter d = 1 + d = 3), as previously described [39]. After removing chimeras, OTUs representing more than 0.005% of the total sequences were retained. A total of 124 OTUs were classified using the reference database silva138, with a pintail quality score 100%. Samples were grouped according to treatments, and normalized using the DESeq2 normalization method. Diversity indices, analysis of significant abundance variation between samples, principal coordinate analysis (PCoA) and all other statistical analyses were realized by SHAMAN [22,40].

### 2.8. Short-Chain Fatty Acid (SCFA) Analysis

Extraction. SCFAs from mice caecum (200 to 400 mg) were extracted in 1 mL of 5 mM aqueous NaOH solution, containing 250 µg/mL of hexanoic acid (caproic acid) as an internal standard (IS). Procedures were performed at 4 °C to protect the volatile SCFAs. The samples were homogenized for 30 min at 4 °C, then centrifuged at 14,000 rpm at 4 °C for 20 min. Standard solutions (ranged from 50 to 200 µg/mL) containing a mixture of acetic acid, propionic acid, isobutyric acid, butyric acid, isovaleric acid and valeric acid were prepared by dissolving the analytical standards mentioned above in 5 mM aqueous NaOH (containing 50 µg/mL of IS).

Samples and standards derivation. A 500 µL aliquot of caecum supernatant or standard solution was transferred into a 10 mL corning glass tube, and 300 µL of water was added to each tube. An aliquot of 500 µL of 2n-propanol/pyridine mixture (3:2, *v*/*v*) and 100 µL of propyl chloroformate (Acros organics, Thermo Fisher Scientific, USA) were subsequently added to the glass tube and vortexed briefly. The derivatization reaction proceeded under ultrasonication for 1 min in an ultrasound bath. A two-step extraction, with n-hexane, was used to extract the derivatives. An aliquot of 300 µL hexane was added to the reaction mixture, and the sample was vortexed for 1 min, followed by centrifugation at 3500 rpm for 5 min. The resulting hexane upper-layer was transferred to a sampling vial containing ~ 10 mg anhydrous sodium sulphate (to remove water traces). The extraction procedure was then repeated by adding 200 µL of n-hexane to the reaction mixture. The resulting n-hexane upper-layer was pooled into two. The first wash was briefly vortexed and diluted with n-hexane before GC–MS analysis.

GC-MS analysis. The derivatized samples were analyzed using a QP 2010 SE GC/MS system (Shimadzu Corporation, Kyoto, Japan) equipped with ZB-5MS capillary column (30 m × 0.25 mm ID, 0.25 μm, Phenomenex). One microliter of derivatives was injected in split mode with a ratio of 20:1, and helium was used as a carrier gas, at a flow rate of 1 mL/min. The injector, detector and ion source temperatures were set at 260, 290 and 230 °C, respectively. The initial column temperature was held at 50 °C for 1 min, then ramped up to 70 °C at a rate of 10 °C /min, from 70 °C to 85 °C at 3 °C /min, from 85 to 110 °C at 5 °C /min, and finally it was increased to 290 °C at a rate of 10 °C /min, and held at this temperature for 8 min. SCFA derivatives were identified by comparing both their MS spectra and retention times with those of the standard mixture, and quantified using calibration curves established with standards.

### 2.9. PICRUS (Phylogenetic Investigation of Communities by Reconstruction of Unobserved States) Analysis

To elucidate the functional potential of microbial communities, we employed PICRUSt2 on our 16S rRNA gene dataset generated by FROGS. This analysis aimed to uncover relevant metabolic pathways, functional genes and enzymatic reactions within microbial populations.

The curated 16S rDNA sequence data underwent processing through the PICRUSt2 pipeline (v2.4) [41]. We focused on MetaCyc pathways, the third-level predictions from PICRUSt2, to examine significant metabolic differences between groups. Pathway abundances were determined using the MetaCyc database, an open-source alternative to KEGG, by mapping EC gene families to pathways.

### 2.10. Statistical Analysis

Statistical analysis was performed using GraphPad Prism software (version 10.01). Data were compared by two-way analysis of variance (ANOVA), followed by Tukey’s multiple comparison tests, and the test data results were expressed as the mean ± standard deviation. A *p*-value less than 0.05 was considered significant.

## 3. Results

### 3.1. Characterization of Grape Seed Polyphenolic Extract (OPC)

BiombalanceBiombalanceThe composition and quantification of the extracted grape seed procyanidins compounds, analyzed by UHPLC-DAD-MS, are presented in Table 1. BB is mainly characterized by procyanidins (23.9 mg/100 mg extract), followed by catechins (12.5 mg) and epicatechins (8.5 mg). Four types of procyanidins were present (B1, B2, B3 and C1), and diverse dimers and trimers of procyanidins. BB also includes acid gallic and epicatechin gallate (1.64 and 0.84 mg/100 mg, respectively) and protocatechuic acid (0.07 mg/100 mg). The UHPLC-DAD chromatogram of the OPC extract is presented in Figure S2. Table 2 presents the polymerization degree of the procyanidins and their quantification. Figure S3 shows the corresponding HPLC-fluorescence chromatogram. The dimers (DP2) and trimers (DP3) were the most represented (19 and 6.1 mg/100 mg, respectively). DP4 to TP7 forms were also present (4.6 mg/ 100 mg), but to a lesser extent.

### 3.2. Antioxidant Capacity of Biombalance™ Extract

Two different assays, ORAC-FL and scavenging of free radicals (DPPH), were selected to determine the antioxidant capacity of BB. The extract showed remarkable oxygen radical scavenging capacity (ORAC), corresponding to 7490.8 μmol Trolox equivalent/g Biombalance™ extract and DPPH radical scavenging IC50 = 24.66 µM TE.

### 3.3. Biombalance™ Extract Alleviates DSS-Induced Colitis Symptoms in Mice

**Figure 1:**
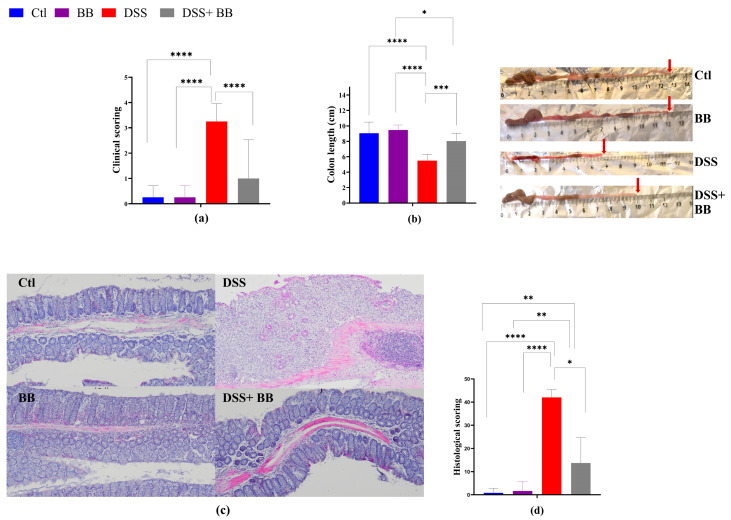
Amelioration of the symptoms of DSS-induced colitis by Biombalance™ supplementation in mice. Ctl: control group, in blue; BB: Biombalance™ extract alone group, in purple; DSS: DSS alone group, in red; DSS+ BB: Biombalance™ + DSS administration group, in grey. (**a**) The disease activity index (DAI) during DSS treatment was calculated based on weight loss, stool consistency and rectal bleeding. (**b**) Colon length. (**c**) Representative images of distal colon histopathological examination results (H&E staining). (**d**) The histological score was calculated from the severity of inflammation, the thickness of inflammatory involvement, epithelial damage and the extent of lesions. (n = 8). * *p* < 0.05, ** *p* < 0.01, *** *p* < 0.001, **** *p* < 0.0001.

Colitis typically manifests with clinical indicators such as weight loss, fecal consistency alterations and bloody stools, parameters used to set the mice disease activity index (DAI) score. DSS treatment induced successful colitis in mice, with a significant escalation in DAI scores compared to the control group (*p* < 0.05) (Figure 1a), validating successful colitis induction. Moreover, DSS significantly reduced the mice’s colon length (*p* < 0.05) (Figure 1b).

DSS mice receiving BB extract (DSS+ BB group) presented a significantly reduced DAI score (*p* < 0.05) (Figure 1a) and an increase in colon length compared with the DSS group (Figure 1b) (*p* < 0.05). As shown in Figure 1d, the colon histopathological score (H&E staining of distal colonic segments) in DSS mice was significantly higher compared to the Ctl group, exhibiting severe inflammatory damage, marked by significant mucosal ulceration, crypt damages, submucosal edema and occurrences of immune cell dysplasia (Figure 1c,d). In contrast, BB treatment substantially improved the histopathological changes observed in the DSS group, alleviating the colonic tissue damage score (*p* < 0.05) (Figure 1c). Moreover, no damage was observed in the colonic mucosa of the control and BB mice groups. These findings indicate that OPCs may reduce colitis symptoms and can enhance colonic damage in mice with colitis induced by DSS. Thus, the BB extract exhibited a beneficial effect on alleviating ulcerative colitis (UC) development in mice.

### 3.4. Biombalance Extract Diminished Production of Proinflammatory Markers in Mice with DSS-Induced Colitis 

**Figure 2:**
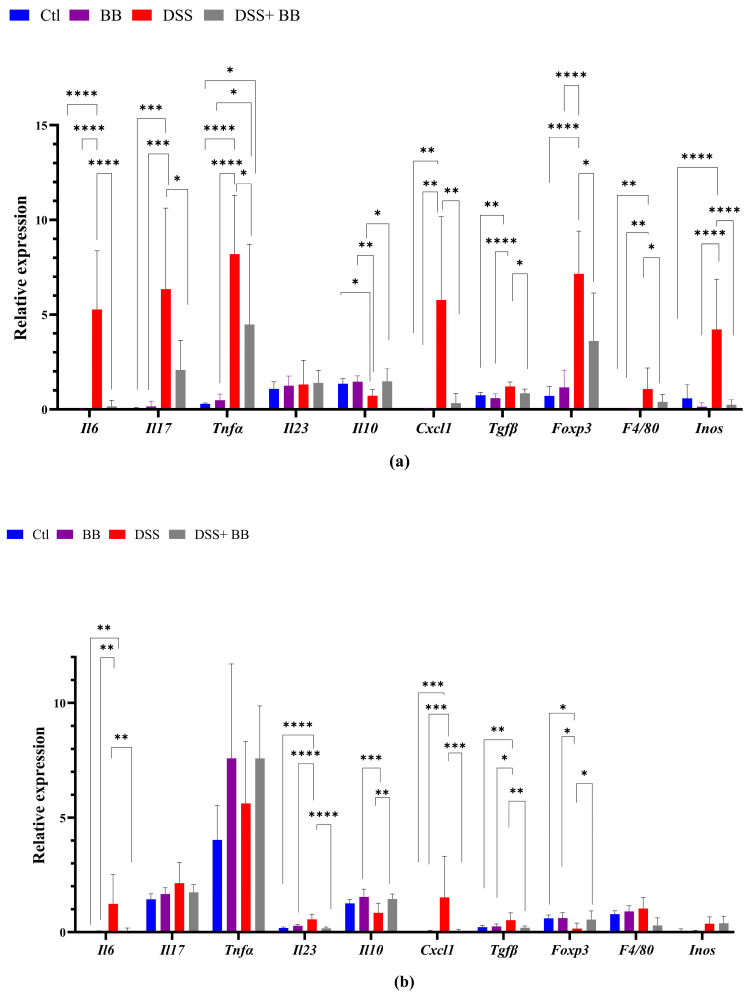

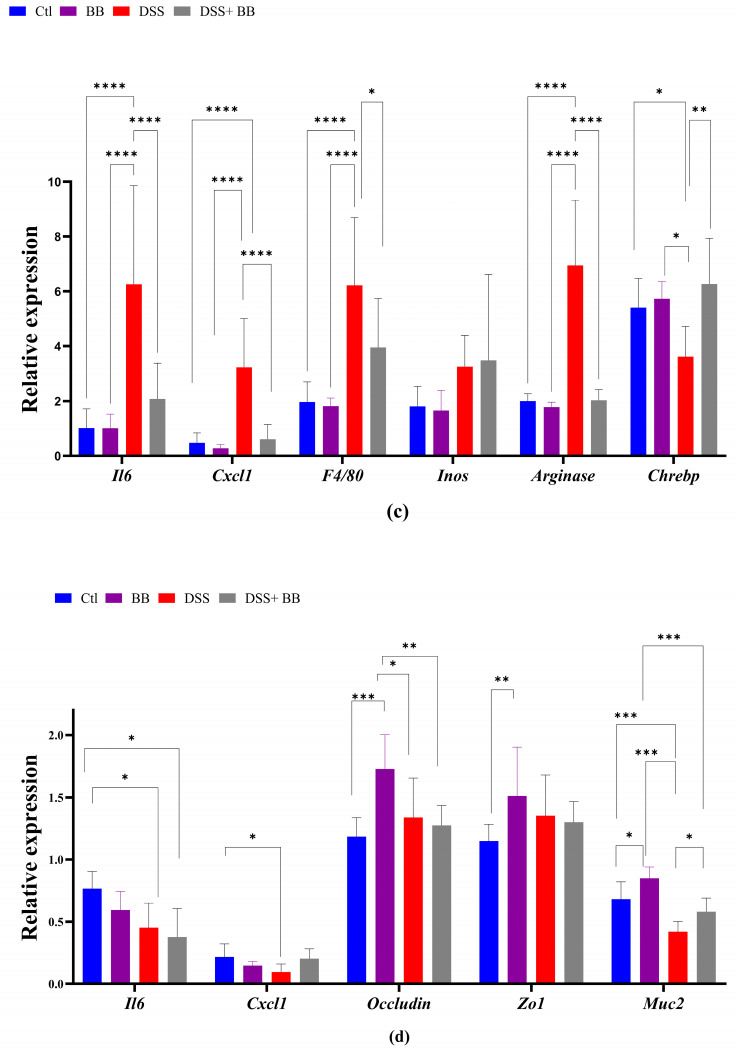
The effect of Biombalance™ on the colon, ileum and liver of DSS-treated mice. Ctl: control group, in blue; BB: Biombalance™ extract alone group, in purple; DSS: DSS alone group, in red; DSS+ BB: Biombalance™ + DSS administration group, in grey. (**a**) The mRNA expression levels of proinflammatory markers in the distal colon. (**b**) The mRNA expression levels of proinflammatory markers in the proximal colon. (**c**) The mRNA expression levels of proinflammatory and homeostasis markers in the liver. (**d**) The mRNA expression levels of proinflammatory and homeostasis markers in the ileum. (n = 8). *p* < 0.05 was identified as the level of statistical significance (* *p* < 0.05, ** *p* < 0.01, *** *p* < 0.001 and **** *p* < 0.0001).

We quantified, using RT-PCR, the level of mRNA expression of various proinflammatory markers in the gut and liver tissues, to observe the anti-inflammatory action of the BB extract. Over-expression of these markers may be involved in the onset and development of UC [42]. In the distal colon, the mRNA expression of the inflammatory cytokines and chemokines IL-6, TNF-α, IL-17 and CXCL1, and the inflammatory mediator’s iNOS, TGF-β, FoxP3 and F4/80, was significantly upregulated in the DSS group compared with the control group (*p* < 0.05). In addition, IL-10 mRNA expression decreased in the DSS group (Figure 2a) compared to the Ctl group. BB treatment normalized the inflammatory response in the distal colon for all the inflammatory markers (*p* < 0.05), except for TNF-α, and restored the anti-inflammatory level of IL-10 (Figure 2a). These results indicate that oral gavage with BB strongly reduced the expression of inflammatory factors in mice with DSS-induced colitis. Given that the proximal and the distal colon have different embryologic origins, and that these regions have distinct transcriptional programmes or cellular processes [43], we also studied the expression of proinflammatory markers and the BB protection effect in the proximal colon. DSS treatment significantly increased the expression levels of the proinflammatory cytokines IL-6, IL-17 and IL-23 compared to the control group, but not TNF-α. Moreover, CXCL1 and TGF-β expressions were significantly increased following DSS administration, but not F4/80. Contrary to the distal colon, the expression of FoxP3 decreased (Figure 2b) in the proximal colon. Moreover, it is essential to note that the level of expression of proinflammatory genes was much lower in the proximal colon than in the distal colon (*p* < 0.05) (Figure 2a,b). In the proximal colon of DSS-treated mice, BB normalized the inflammatory response induced by DSS, reducing the expression level of proinflammatory genes to the same level as the Ctl group, including F4/80 (Figure 2b). However, the expression of IL-17 in the BB-DSS group was not decreased compared to the DSS group. We only observed a trend for IL-17. Depending on the part of the colon analyzed, the quantity and profile of proinflammatory markers were not identical. As DSS treatment can affect the entire gastrointestinal tract and potentially other organs, we evaluated the effect of DSS on proinflammatory markers in the ileum and liver. Contrary to the colon, mRNA expressions of IL-6 and CXCL1 in the ileum were slightly but significantly reduced in the DSS group, instead indicating migration of immune cells toward the colon, the principal site of inflammation in this model (Figure 2d). Regarding the liver, the DSS group presented an increase in the mRNA expression of IL-6, CXCL1, F4/80 and arginase genes, while this expression was significantly reduced in the DSS+ BB group (Figure 2c), reaching the level of the Ctl group, except for the F40/80 gene expression. These results indicate that the liver inflammation caused by the DSS treatment was strongly attenuated with BB (Figure 2c). However, DSS treatment did not significantly increase *iNOS* mRNA expression. As intestinal and liver inflammatory contexts may affect liver metabolism, we analyzed the mRNA expression of a central regulator of metabolism: the transcription factor carbohydrate response element binding protein (ChREBP). Its expression in the liver was significantly decreased in the DSS group compared to the Ctl group, and increased in the DSS+ BB group, reaching the control level (*p* < 0.01). In this context, BB could be of interest, because ChREBP activation is associated with improved insulin sensitivity in the adipose tissue and liver in mice [44].

### 3.5. Involvement of Biombalance™ in Oxidative Stress and Gut Barrier Integrity

**Figure 3:**
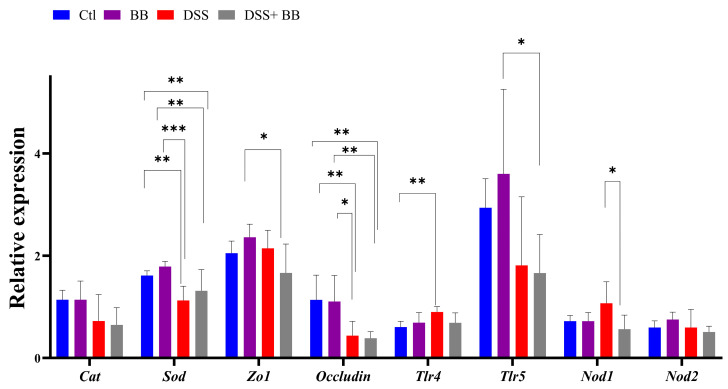
The effects of Biombalance™ supplementation on oxidative stress and gut barrier integrity in the proximal colon in DSS-treated mice. Ctl: control group, in blue; BB: Biombalance™ extract alone group, in purple; DSS: DSS alone group, in red; DSS+ BB: Biombalance™ + DSS administration group, in grey. (n = 8). *p* < 0.05 was identified as the level of statistical significance (* *p* < 0.05, ** *p* < 0.01, *** *p* < 0.001).

We studied the expression of other homeostatic markers in the proximal colon because, in the distal colon, the expression of several genes appeared not to be induced with the DSS treatment. Compared to the Ctl group, the expression of the oxidative stress marker CAT was not modified, but that of SOD was decreased in the DSS group (*p* < 0.01). However, SOD expression was not increased in the DSS+ BB group (Figure 3). Tight-junction proteins such as ZO1 and occludin are essential for intestinal barrier maintenance [45]. DSS treatment only significantly decreased the mRNA expression of occludin, but the BB extract did not restore its expression (Figure 3). We also analyzed the mRNA expression of the TLR4, TLR5, NOD1 and NOD2 genes. NOD1 expression increased in the DSS group compared to the DSS+ BB group. TLR4 expression also increased in the DSS group, but compared to the control, a decreasing trend was observed in the DSS+ BB group (Figure 3). Further, NOD2 expression was identical in all groups, and TLR5 expression was only increased in the BB group (Figure 3). In the ileum, compared to the control, MUC2 expression was increased in the BB group (*p* < 0.05) and reduced in the DSS group (*p* < 0.001), and in the DSS+ BB group, MUC2 expression was increased compared to the DSS group (*p* < 0.05) (Figure 2d). Moreover, ZO1 and occludin expressions were also increased in the BB group compared to the control (ZO1) and to the other groups (occludin) (Figure 2d). These results may indicate a beneficial effect of the BB extract on the integrity of the ileum epithelium.

### 3.6. Biombalance™ Supplementation Influences Caecum SCFA Generation

**Figure 4:**
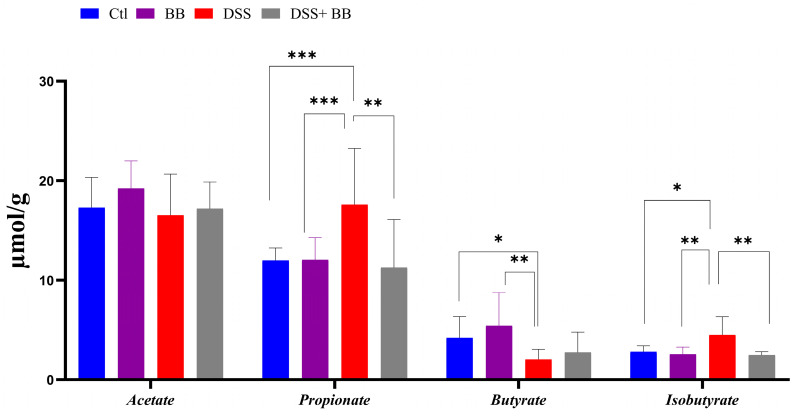
Biombalance™ extract supplementation influenced caecum SCFA generation in DSS-treated mice. Ctl: control group, in blue; BB: Biombalance™ extract alone group, in purple; DSS: DSS alone group, in red; DSS+ BB: Biombalance™ + DSS administration group, in grey. (n = 8). *p* < 0.05 was identified as the level of statistical significance (* *p* < 0.05, ** *p* < 0.01, *** *p* < 0.001).

Intestinal bacteria produce short-chain fatty acids (SCFAs), especially butyrate, exerting beneficial effects on intestinal health, and can have beneficial effects on IBD [46]. Compared to the control, DSS significantly increased the propionate and isobutyrate levels and decreased butyrate formation in caeca (Figure 4). However, propionate and isobutyrate content significantly decreased in the DSS+ BB group, reaching the level of the control group (Figure 4). On the other hand, butyrate was increased in the DSS+ BB group compared to the DSS group, but not significantly (Figure 4).

### 3.7. Effects of Biombalance™ on Gut Microbiota in Mice with DSS-Induced Colitis

**Figure 5:**
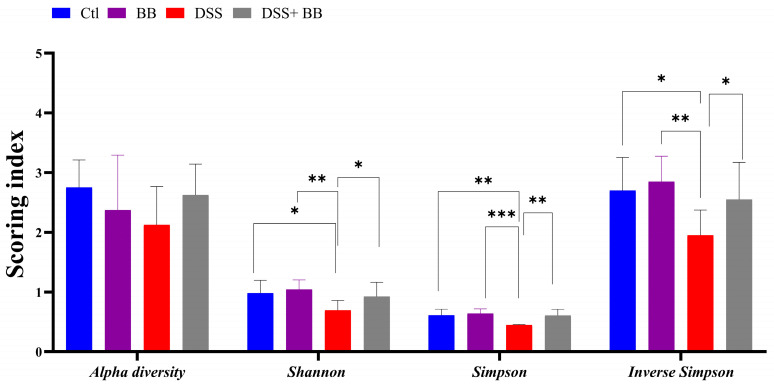
Effects of Biombalance™, DSS and DSS administered with Biombalance™ on microbiota alpha diversity, Shannon diversity, Simpson diversity and inverse Simpson diversity. Ctl: control group, in blue; BB: Biombalance™ extract alone group, in purple; DSS: DSS alone group, in red; DSS+ BB: Biombalance™ + DSS administration group, in grey. Data are expressed as the mean ± SEM. (n = 8). *p* < 0.05 was identified as the level of statistical significance (* *p* < 0.05, ** *p* < 0.01, *** *p* < 0.001).

The gut microbiota is involved in several critical physiological functions in the host, including immune and gut homeostasis. Thus, the impact of microbiota alteration has been extensively studied in recent years in the context of inflammatory diseases, particularly in the context of inflammatory gut disease. The Illumina MiSeq platform was used to analyze the gut microbiota changes associated with DSS-induced colitis, and associated or not with Biombalance™ treatment. A total of 485718 sequencing reads for the fecal microbiota were analyzed using GALAXY [39] and SHAMAN [40]. Alpha diversity reflects abundance and homogeneity in a community. Our results show that the alpha diversity was not significantly different between the four groups (Figure 5). Nevertheless, differences were observed in other indices. The Shannon diversity index is influenced by species richness and evenness in samples, and thus can reflect the species homogeneity of a bacterial community [47]. A higher Shannon index indicates greater diversity and even distribution within the microbiota community. This index was significantly lower in the DSS group compared to the Ctl group (*p* < 0.01). This effect was significantly counteracted by the administration of BB in mice treated with DSS (*p* < 0.05) (Figure 5). Both Simpson and Inverse Simpson indices assign more significance to the abundance of the most dominant species present in a community, and provide a comprehensive measure of microbial diversity, incorporating both species richness and evenness, with higher values typically indicating a more balanced and diverse microbiota [48]. These indices were significantly decreased by the DSS treatment, and increased, returning to the level of the Ctl group, following BB treatment in the BB+ DSS group (*p* < 0.01) (Figure 5).

**Figure 6:**
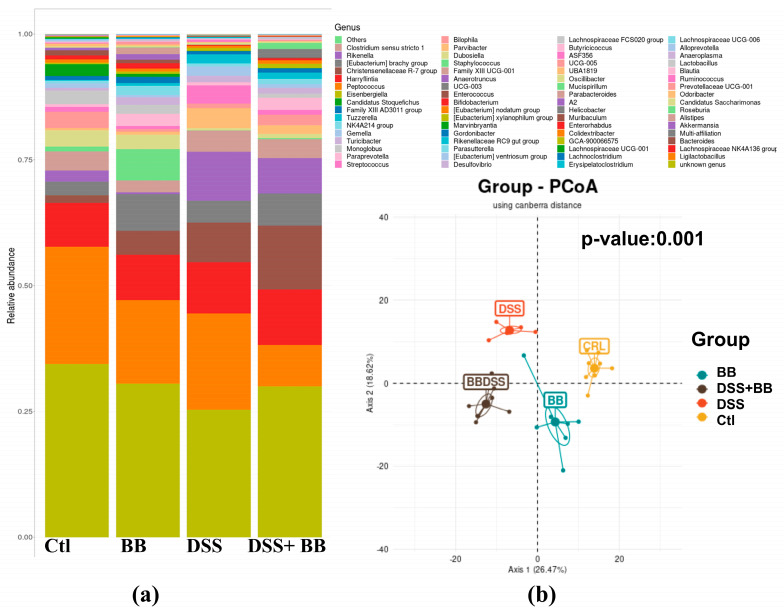
Taxonomic composition of the gut microbiota under different types of treatments at the genus level (n = 8). (**a**) Barplot of the proportions of various taxa in different conditions. (**b**) Principal coordinate analysis using Canberra distance; Axis 1: 26.47%, Axis 2: 18.62%, *p*-value: 0.001.

**Figure 7:**
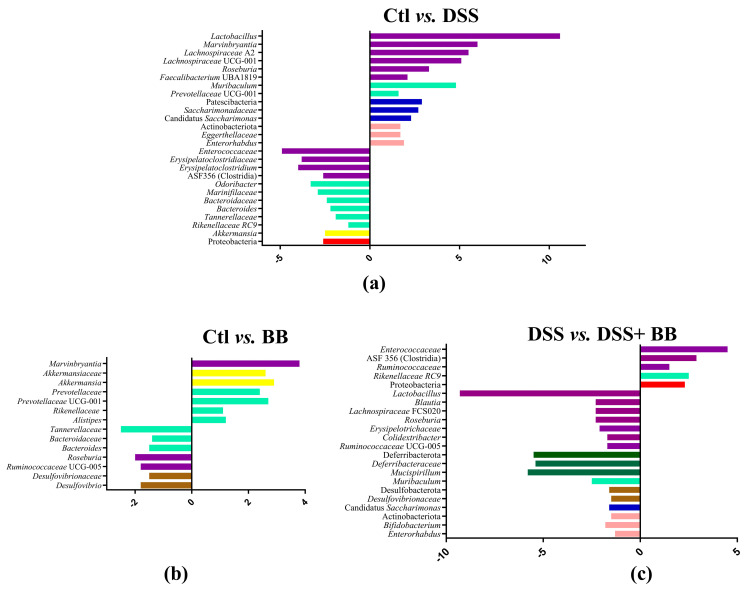
Log2 fold changes in predicted microbiota abundance for different experimental contrasts. (**a**) Ctl vs. DSS, (**b**) Ctl vs. BB and (**c**) DSS vs. DSS+ BB. Only bacteria showing significant differences in the comparisons (*p* < 0.05) and an abundance equal to or greater than 0.2% are presented. The bars’ colours represent bacteria belonging to different phyla. Purple: Bacillota; light green: Bacteroidota; dark blue: Patescibacteria; pink: Actinobacteriota; yellow: Verrucomicrobia; red: Proteobacteria; brown: Desulfobacterota; dark green: Deferribacterota.

Subsequently, we performed principal coordinate analysis (PCoA) of genus levels to evaluate the gut microbiota compositions between groups (Figure 6b). PCoA showed a significant separation among the four groups, Ctl, BB, DSS and DSS+ BB (*p* = 0.001), indicating four different clusters, considering their microbiota composition. Analysis of microbiota composition at the phylum level revealed that Proteobacteria, a phylum including some proinflammatory genders, were increased in the DSS group compared to Ctl, contrary to Patescibacteria and Actinobacteriota, which were decreased in the DSS group (Figure 7a). Interestingly, using BB decreased Proteobacteria while increasing Actinobacteriota and other minority phyla (Figure 7c and Figure S4). At the family level, *Lachnospiraceae*, *Muribaculaceae*, *Lactobacillaceae* and *Bacteroidaceae*, were the families that were represented the most in the four groups (Figure S5). DSS treatment decreased the *Saccharimonadaceae* (from *Patescibacteria* phylum) and *Eggerthellaceae* families and increased the *Enterococcaceae*, *Bacteroidaceae*, *Tannerellaceae* and *Erysipelatoclostridiaceae* families, which can be implicated in gut inflammatory diseases. At the genus level, DSS-treated mice revealed substantial shifts in gut microbiota composition, mirroring the dysbiosis characteristic of inflammatory bowel disease. Comparing the Ctl and DSS groups (Figure 7a), we retrieved many significant genus variations (<to 0.2%) in the DSS group (*p* < 0.05). These mice exhibited a notable proliferation of *Bacteroides*, *Akkermansia*, ASF 356 (Clostridia), *Odoribacter*, *Enterococcus* and some minority potential pathogenic bacteria (*Staphylococcus*) (Figure 7a). This expansion aligns with previous research in DSS models, although the observed increases in *Odoribacter* and ASF356 are less commonly reported. Concurrently, beneficial bacteria with possible anti-inflammatory properties, such as *Lactobacillus*, Candidatus *Saccharimonas*, *Muribaculum*, *Roseburia* and various *Lachnospiraceae* members, showed significant declines in the DSS group. This reduction in protective commensal bacteria in the DSS-treated mice parallels observations in human IBD patients and other DSS models [48]. The BB group, when compared to the control, displayed a unique microbial profile characterized by increased levels of *Roseburia*, *Bacteroides*, *Ruminococcaceae* UCG-005 and *Desulfovibrio*, and reductions in *Alistipes*, *Akkermansia* and *Prevotellaceae* UGC-001 (Figure 7b). These alterations suggest that BB alone can modulate gut microbiota, potentially fostering the growth of beneficial bacteria.

More remarkably, the comparison between DSS-treated mice receiving BB and those receiving DSS alone revealed a significant restoration of potentially beneficial bacteria (*Lactobacillus*, *Muribaculum* and various *Lachnospiraceae* genera, as well as *Roseburia*, *Muscispirillum*, Candidatus *Saccharimonas*, *Bifidobacterium*, *Desulfovibrio* and *Colidextribacter*) with a decrease in *Rikenellaceae* RC9, Clostridia ASF356 and several potentially harmful minority genera (including *Enterococcus*, *Staphylococcus*, and *Streptococcus*) (Figure 7c, and Figures S6 and S7).

These findings indicate that BB treatment may help to re-establish the microbial equilibrium disrupted by DSS, potentially promoting commensal bacteria and suppressing opportunistic pathogens. The observed shift towards a more balanced microbial community in BB-administered DSS mice resembles effects seen with other efficacious interventions in DSS colitis models. On the other hand, BB can be involved in the restoration of the gut bacterial cross-feeding equilibrium that is disrupted in IBD, including the modulation of different metabolites, such as SCFAs H_2_ and SH_2_ [49].

### 3.8. PICRUSt Results

**Figure 8:**
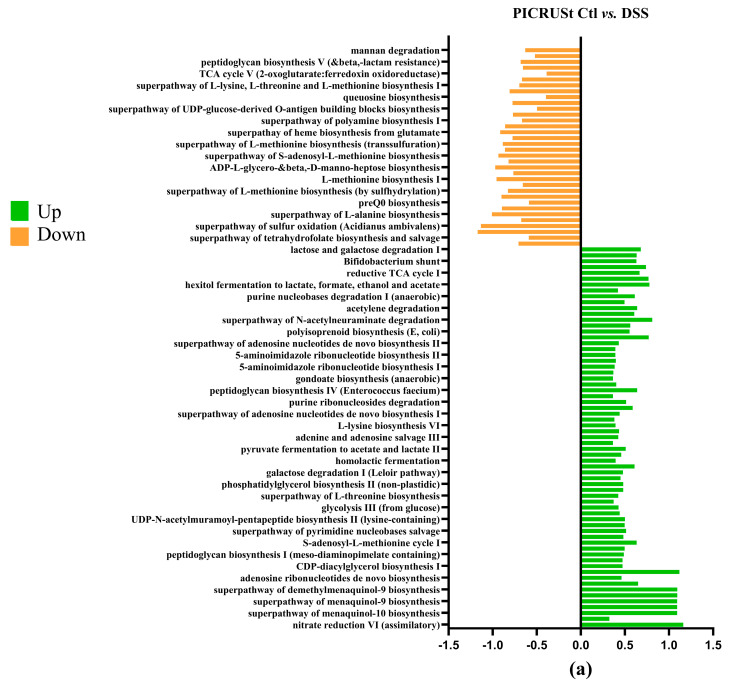

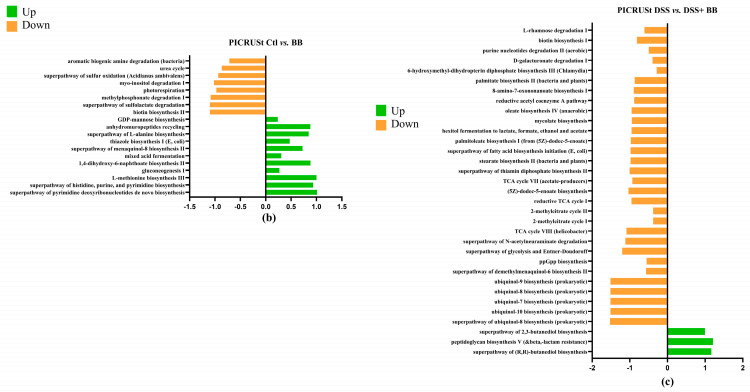
Log2 fold changes in predicted microbiota abundance using (KEGG) for different experimental contrasts (n = 8). (**a**) Ctl vs. DSS, (**b**) Ctl vs. BB and (**c**) DSS vs. DSS+ BB. Green—significantly increased, and orange—significantly decreased (*p* < 0.05).

To investigate microbial communities’ metabolic capabilities and functional genes, we employed PICRUSt2 analysis of 16S rRNA gene sequences. This computational approach yielded several important insights: the study identified 273 distinct KEGG Orthology (KO) codes, providing a comprehensive overview of the predicted functional repertoire within these microbial communities. These findings highlight that the complex modification of microbiota composition induced by the DSS treatment (Figure 8a) is directly related to changes in metabolic pathways, predicted by PICRUSt analysis [50] as impacts on sugar metabolism, amino acid biosynthesis, purine nucleotide biosynthesis and many other functions [49]. The DSS+ BB treatment group had significant modifications compared to the DSS group (Figure 8c). For example, the methyl citrate (2-MCC) cycle is necessary to metabolize and detoxify propionate by pyruvate oxidation [51]. The BB treatment significantly increased this pathway compared to the DSS group, which may explain the decrease in the SCFA dosage of propionate (Figure 4). Interestingly, BB also increased the antioxidant superpathway of ubiquinol biosynthesis, including ubiquinol-7, ubiquinol-8, ubiquinol-9 and ubiquinol-10 (Coenzymes Q). CoQ is essential in several aspects of cellular metabolism, shielding membrane lipids from oxidative stress [52]. These antioxidant capacities linked to the gut microbiota may help to reduce the effects of DSS by decreasing inflammation and oxidation [53].

## 4. Discussion

The present study demonstrates the potential of Biombalance™, a grape seed-derived extract rich in procyanidins, in mitigating DSS-induced colitis in mice. Our findings reveal the multifaceted effects of this extract on various aspects of intestinal health, including clinical symptoms, histopathological changes, inflammatory markers, oxidative stress, gut barrier integrity and gut microbiome alteration/dysbiosis. The major components of BB are catechins, epicatechin and B-type procyanidin dimers (representing nearly 80% of extracted procyanidins), followed by procyanidin trimers (11.4%) and tetramers (5.7%). Differences in the composition of monomers and the degree of polymerization of OPCs in plant extracts contribute to variation in the profile and effect of procyanidins [26]. These differences could also affect plant extracts’ antioxidant capacity and free radical scavenging, without forgetting their impact on the microbiota. Indeed, the literature describes how the polyphenolic profile and biological activities of the same plant extract depend on the quality of the raw material and extraction methods [54].

BB exhibited remarkable antioxidant properties, with an oxygen radical scavenging capacity (ORAC) of 7490.8 μmol Trolox equivalent/g extract. This potent antioxidant activity is likely due to the high content of monomeric and dimeric proanthocyanidins in the extract’s profile. The specific polyphenolic profile of BB regarding procyanidin monomers (catechins and epicatechins) and types of procyanidin dimers (B- and C-type) could be linked to its high antioxidant capacity. Indeed, those molecules have been described in the literature to have a more potent antioxidant effect than other oligomers [55], and work through combined oxidation inhibition [56]. The antioxidant capacity of BB is interesting, considering that IBD is a significant oxyradical-overload disease [57].

In addition to their antioxidant effects, catechins and dimeric B-type procyanidins can provide anti-inflammatory benefits, due to their ability to reduce *NF-κB* signalling [58], thus reducing the production of proinflammatory cytokines and chemokines in different types of cells, such as macrophages and dendritic cells. In the present study, we observed the anti-inflammatory effect of BB in the colon and liver of DSS-treated mice. Comparing with a study using pycnogenol in IBD [59], our study confirms that the polyphenolic profile of a plant extract is of high importance, even if the content of polyphenols and OPCs is high.

In the immune system, iNOS-derived NO is crucial for destroying pathogens, but excessive production can lead to oxidative damage [60]. In our study, exacerbated *iNos* expression was retrieved principally in the distal colon of the DSS group, and the BB treatment completely attenuated these *iNos* expressions, indicating that OPCs can improve cellular endogenous antioxidant mechanisms [15,60]. In the liver, DSS or BB treatment did not significantly modify *iNos* expression, but the DSS treatment significantly increased arginase expression, and the BB treatment decreased it. Garate-Carrillo et al. [61] demonstrated that oxidative stress can increase arginase activity. Epicatechin’s antioxidant properties can inhibit arginase activity, and thus mitigate the effects of oxidative stress [61]. Epicatechin in BB can explain the decrease in arginase observed in the liver, among other observations. Gene expression analysis revealed significant changes in key inflammatory and barrier function genes following BB treatment.

We found that in colon tissues, compared with the DSS group, BB supplementation decreased the expression of proinflammatory markers (IL-6, TNF-α, IL-17 and CXCL1) and inflammatory mediators (TGF-β, FoxP3 and F4/80), and we also observed that the damage caused by the DSS impacted the distal colon more than the proximal part [62]. Chemokine CXCL1 can have a pivotal role in promoting neutrophil infiltration in colitis. In fact, CXCL1−/− mice exhibited a great reduction in colitis after DSS treatment [63]. Interestingly, in our study, BB strongly reduced the expression of CXCL1 in the colon and liver. Concerning inflammatory mediators, previous studies have proposed the role of TGF-β signalling in gut inflammation [64]. Interestingly, BB treatment decreased the expression level of *TGF-β* in the colon segments. In addition, the expression of *F4/80*, a well-known marker of murine macrophage populations, increased by the DSS treatment, decreased in the colon and liver of the BB group, suggesting the role of BB in preventing the further recruitment of macrophages in colitis [65].

Notably, we observed a marked upregulation of *IL-10* expression in the colon of BB-treated mice. IL-10 is a crucial anti-inflammatory cytokine that plays a central role in intestinal homeostasis and has been implicated in IBD pathogenesis [66]. The increase in *IL-10* expression correlated strongly with the abundance of certain beneficial bacteria, particularly *Lactobacillus* species, suggesting a potential microbiota-dependent mechanism for IL-10 induction [67].

Our study also revealed the systemic effects of BB treatment, particularly on liver inflammation. The reduction in hepatic inflammatory markers suggests that the extract’s benefits extend beyond the gastrointestinal tract, potentially protecting against extra-intestinal manifestations of IBD [68]. ChREBP could play a protective role against oxidation and inflammation, ChREBP-deficient macrophages show increased inflammatory responses and oxidative stress-induced apoptosis [69]. In our study, the DSS treatment significantly decreased *ChREBP* expression, and the BB treatment brought the expression back to normal. Other polyphenols, like dietary polyphenol curcumin, positively affected *ChREBP* expression [70]. Our study is the first to describe the positive impact of OPCs extract on liver *ChREBP* expression in a DSS model. Several preclinical and clinical studies have suggested that plant extracts rich in polyphenols could alter the composition of the gut microbiota. In the context of IBD, it has been demonstrated that the gut microbiota is highly altered. Researchers have suggested that the breakdown of the gut microbiome and the establishment of dysbiosis have become key processes in the development of IBD. Some microbiota groups known to possess anti-inflammatory properties have been shown to be decreased in IBD patients, at the expense of proinflammatory bacteria. Notably, it has been suggested that the *Proteobacteria* phylum could be essential in initiating chronic inflammation in IBD [1]. One of the most striking findings of our study was the significant modulation of the gut microbiota by Biombalance™ administration. The DSS+ BB group showed higher values for various diversity measures (Shannon, Simpson and inverse Simpson indices) than the DSS group. This indicates that the extract likely encourages the development of a more varied and well-balanced microbial ecosystem. This is particularly important, given the growing evidence linking reduced microbial diversity to IBD [71].

The principal coordinate analysis (PCoA) revealed distinct clustering of the microbiota profiles in the BB-treated group, indicating a shift towards a healthier microbial composition and/or a functional equilibrium. This shift was characterized by increased beneficial bacteria, such as *Lactobacillus*, *Muribaculum* and *Roseburia*, alongside decreases in potentially harmful bacteria, like *Enterococcus* and *Staphylococcus*. These changes in microbial composition are consistent with those observed in other studies using prebiotic interventions in obesity models [22].

The observed increase in *Lactobacillus* is significant. *Lactobacillus* species play crucial roles in maintaining gut health by enhancing barrier function, modulating immune responses, and competing with pathogenic bacteria [67,72]. The ability of BB to promote the growth of *Lactobacillus* suggests that it may act as a prebiotic, selectively stimulating the development of these beneficial microbes.

The modifications concerning short-chain fatty acid (SCFA) production play an important role in gut homeostasis. SCFAs, especially butyrate, are known to play a crucial role in maintaining intestinal health and to have positive effects on IBD [73]. Although BB does not significantly affect butyrate, it counterbalances the effect of DSS on SCFAs by decreasing the level of two other SCFAs: propionate and isobutyrate. Isobutyrate, known as DSS colitis marker [74], was significantly decreased by BB treatment. Moreover, propionate can be significantly increased in DSS models [75]. BB counterbalanced this augmentation [76]. This observation could be linked to the effect of BB on gut microbiota. Indeed, BB treatment decreased some propionate and isobutyrate producer groups, like *Rikenellaceae* and ASF256 [77,78], that were increased by the DSS treatment.

Interestingly, the PICRUSt results showed that ubiquinol pathways were significantly increased in the DSS+ BB group compared to the DSS group. Ubiquinols are the reduced form of ubiquinones like CoQ7 to CoQ10 (Co-enzyme Q), and both have strong antioxidative capacities [79]. Their role in bacteria is to regulate energy metabolism, gene expression and the prevention of oxidative stress [80]. This predicted over-expression of ubiquinol pathways from the gut microbiota could demonstrate a selection of bacteria that are more resistant to the oxidative environment caused by DSS. Other studies have shown that the microbiota of non-obese individuals has a more significant proportion of ubiquinol biosynthesis-related pathways than that of obese individuals [81]. No study has explained whether bacterial ubiquinols can be secreted to affect host cells positively. It is known that bacterial extracellular vesicles (BEVs) have a multifaceted role in mediating host–microbe interactions across various physiological conditions [82,83]. Given that ubiquinols are membrane fat-soluble metabolites, we can innovatively speculate that BEVs have potential as vehicles for transporting CoQ to intestinal cells. Furthermore, the administration of ubiquinol in a rat model of enteropathy induced by radiation had a gastroprotective effect, regulated the expression of proinflammatory cytokines, inhibited the expression of NF-κB and restored gut microbiota dysbiosis [84]. In another study, the administration of CoQ10 in rats modified the composition of gut microbiota and increased the generation of molecular hydrogen [85].

Physiological H_2_ concentrations might protect the mucosa from oxidative insults [86,87]. In addition, H_2_ can regulate various signalling pathways, such as Nrf2 (antioxidant response regulator), PI3K/Akt, JAK2/STAT3 and NF-κB (inflammatory responses) [88]. The most abundant bacteria responsible for H_2_ production in the colon are *Ruminococcus*, *Roseburia*, *Blautia*, *Lachnospiraceae*, *Bacteroides* and *Clostridium* spp. [89]. In our study, the first four of these bacteria were increased in the DSS+ BB group, signalling H_2_ production in the colon. The interaction between H_2_-producing and H_2_-consuming bacteria is essential for maintaining colon homeostasis. Hydrogenotrophic (H_2_–utilizing) microbes, such as *Mucispirilum* and sulphate-reducing bacteria (SRB), can mediate the removal of excess H_2_-reducing sulphate with H_2_ to form H_2_S [90,91]. SRB are ubiquitous members of the mammalian colon, and represent a significant source of gut and circulating H_2_S (>65% of plasma-free H_2_S) [92]. *Desulfovibrionaceae* members, such as *Desulfovibrio*, are the most frequent SRB present in the colon. In our study, these bacteria were increased in the DSS+ BB group compared to the DSS group. Functionally, the H_2_S endogenously produced by SRB can react with ROS, like superoxide (O_2_^•−^), and upregulate various antioxidant systems, such as the Nrf2 pathway [93]. Moreover, H_2_S possesses an anti-inflammatory role, stimulating the JAK2/STAT3 signalling pathway, promoting the polarization of macrophages from the M1 to M2 type, reducing leukocyte migration to the site of injury and enhancing neutrophil apoptosis [91,92,93]. In the continuum with these cross-feeding interactions, the produced H_2_S can, in turn, be used by H_2_S consumers, such as *Roseburia* and *Blautia* [50]; these bacteria were also increased in our study when BB was administered. The Marcelino study [50] observed that the H_2_S balance was disrupted in Crohn’s disease, resulting in a higher H_2_S producer-to-consumer ratio.

An equilibrium between cross-feeding interactions is essential for host homeostasis, and controlling these interactions can represent an important target of future research.

## 5. Conclusions

Our study’s comprehensive approach to examining the effects of BB on multiple aspects of colitis pathogenesis provides a more holistic understanding of its beneficial health effects. Integrating microbiome analysis with gene expression and metabolite profiling offers unique insights into the complex interactions between the gut microbiota, host immune responses and intestinal barrier function in the context of IBD [87,90].

In addition to the direct effect of polyphenols as antioxidants, a synergic indirect mechanism of action takes place through their capacity to regulate gut microbiota composition [94], which ultimately can also, in turn, activate various antioxidative processes, such as promoting radical scavengers and the expression of cellular antioxidant pathways, and regulating inflammatory processes. In this context, we must not forget the role that bacterial metabolites, such as ubiquinones, H_2_ and H_2_S, can play.

The unique composition of Biombalance™, with its mix of low- and high-molecular-weight OPCs, may contribute to its superior efficacy compared to other polyphenol extracts. These findings highlight the potential of Biombalance™ as a novel prebiotic with high antioxidant potential. Future studies can focus on elucidating the specific molecular targets of OPCs in Biombalance™ and exploring the potential of this prebiotic against other health issues.

## Figures and Tables

**Table 1:** Composition and quantification of procyanidins in Biombalance™ extract analysed by UHPLC-DAD-MS.

Time (min)	Weight (Da)	[M − H]^−^	Fragment Ions in Negative Mode	Structural Hypothesis	Biombalance™ ($)
2.0	170	169	125	Gallic acid * ^(a)^	1.64 ± 0.01
3.4	154	153	109	Protocatechuic acid * ^(b)^	0.070 ± 0.001
4.6	578	577	425, 407, 289	Procyanidin dimer B1 * ^(c)^	5.55 ± 0.02
4.9	578	577	425, 407, 289	Procyanidin dimer B3 * ^(c)^	2.22 ± 0.03
ok5.2	290	289	245, 205	Catechin * ^(d)^	12.49 ± 0.03
5.4	866	865	577	Procyanidin trimer ** ^(c)^	1.14 ± 0.02
5.5	578	577	425, 407, 289	Procyanidin dimer ** ^(c)^	0.97 ± 0.03
5.8	578	577	425, 407, 289	Procyanidin dimer B2 * ^(c)^	4.57 ± 0.04
6.1	290	289	245, 205	Epicatechin * ^(e)^	8.52 ± 0.06
6.3	730	729	577, 559, 451, 289	Procyanidin dimer gallate ** ^(c)^	1.80 ± 0.06
6.5	866	865	577, 289	Procyanidin trimer C1 ** ^(c)^	1.28 ± 0.02
6.7	578	577	425, 407, 289	Procyanidin dimer ** ^(c)^	0.88 ± 0.01
6.8	730	729	577, 559, 451, 289	Procyanidin dimer gallate ** ^(c)^	3.86 ± 0.08
7.4	882	881	729, 577	Procyanidin dimer digallate ** ^(c)^	1.02 ± 0.02
7.6	442	441	289	Epicatechin gallate * ^(f)^	0.84 ± 0.02

Legend: ($): Contents in mg/100 mg (n = 3 repetitions); *: Structural hypothesis confirmed by commercial standard; **: Attempt to identify compound from molar mass and fragment ions; Quantification methods: (a) quantified in gallic acid equivalent at 280 nm; (b) quantified in protocatechuic acid equivalent at 260 nm; (c) quantified in dimeric procyanidin equivalent B2 at 280 nm; (d) quantified in catechin equivalent at 280 nm; (e) quantified in epicatechin equivalent at 280 nm; (f) quantified in epicatechin gallate equivalent at 280 nm.

**Table 2:** Quantification of procyanidins according to their degree of polymerization in Biombalance™ extract.

Retention Time (min)	Degree of Polymerization (DP)	Contents in mg/100 mg Eq. Epicatechin (n = 3 Repetitions) Biombalance™
7.0	Monomers (DP1)	23.39 ± 0.44
12.3	Dimers (DP2)	18.92 ± 0.28
15.8	Trimers (DP3)	6.1 ± 0.15
18.5	Tetramers (DP4)	3.04 ± 0.02
20.6	Pentamers (DP5)	1.05 ± 0.006
22.3	Hexamers (DP6)	0.46 ± 0.01
23.7	Heptamers (DP7)	0.09 ± 0.004
25.0	Octamers (DP8)	<LQ *

* <LQ: content below limit of quantification.

## Data Availability

The article’s material includes the original contributions presented in this study. Further inquiries can be directed to the corresponding author.

## Supplementary Materials

The following supporting information can be downloaded at https://www.mdpi.com/article/10.3390/antiox14030305/s1: Figure S1: Standard mice diet composition; Figure S2: UHPLC-DAD chromatogram of the OPC extract; Figure S3: Separation of procyanidins according to their degree of polymerisation by HPLC-fluorescence; Figure S4: Log2 fold changes in phylum abundance for different experimental contrasts, (a) Ctl vs DSS, (b) Ctl vs BB, (c) DSS vs DSS+ BB); Figure S5: Log2 fold changes in families abundance for different experimental contrasts, (a) Ctl vs DSS, (b) Ctl vs BB, (c) DSS vs DSS+ BB; Table S1: Colon histological scores; Table S2: RT-qPCR primer sequences.

## Abbreviation

Arg1	arginase 1
CAT	catalase
ChREBP	carbohydrate-responsive element-binding protein
CXCL-1	C-X-C Motif Chemokine Ligand 1
DPPH	2,2-Diphenyl-1-picrylhydrazyl
DSS	Dextran Sulphate Sodium
F4/80	(Adgre1) adhesion G protein-coupled receptor E1
FoxP3	forkhead box P3
Hprt	hypoxanthine phosphoribosyltransferase
IL-6	interleukin 6
IL-10	interleukin 10
IL-17	interleukin 17
IL-23	interleukin 23
iNOS	inducible nitric oxide synthase
LC-MS/MS	liquid chromatography–tandem mass spectrometry
MALDI	matrix-assisted laser desorption ionization
MUC2	mucin 2
NMR	nuclear magnetic resonance
NO	nitric oxide
NOD-1	nucleotide-binding oligomerization domain-containing protein 1
NOD-2	nucleotide-binding oligomerization domain-containing protein 2
Ocln	occludin
PB1	procyanidin dimer B1
PB2	procyanidin dimer B2
PC1	procyanidin trimer C1
SOD	superoxide dismutase
TGF-β	transforming growth factor-beta
TLR4	toll-like receptor 4
TLR5	toll-like receptor 5
TNF-α	tumour necrosis factor α
ZO-1	zonula occludens-1
