# Supplementary material for: Biombalance™, an Oligomeric Procyanidins-Enriched Grape Seed Extract, Prevents Inflammation and Microbiota Dysbiosis in a Mice Colitis Model
Source: Antioxidants (Basel). 2025 Mar 1;14(3):305. doi: 10.3390/antiox14030305 (PMC11939601; doi:10.3390/antiox14030305)
Supplement: Supplementary file 1 [file antioxidants-14-00305-s001.zip › S1.pdf]

SAFE® A04

Definition

Complete maintenance diet for rats, mice and hamsters.

Product Purpose

Diet for adult and maintenance animals.
To be used within the context of experimental protocols.
Does not contain alfalfa and its byproducts.

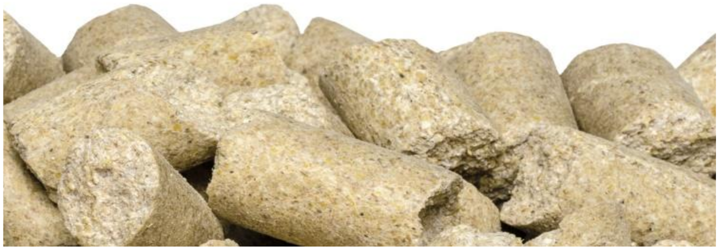

Picture indicative only

Directions for Use

DISTRIBUTION

Period

After weaning and adult.

Method

- Ad libitum or rationed according to experimental protocols.
- Remove from the packaging and place directly in the cage feeder or on the cage floor.
- Keep fresh water always available.

DAILY CONSUMPTION

Rats 18 to 25 g, mice 3 to 6 g, hamsters 8 to 12 g.

STORAGE

Store in a clean, dry and cool place, protected from light.

SHELF-LIFE from the date of production

Paper bag or plastic pouch = 12 months
Vacuum packed = 24 months

Irradiation

Possible doses: Minimum 10, 25 or 40 kilograys.

Product Form

PELLETS	Mean
Diameter	16,4 mm
Crushing resistance	22,7 kgf/cm²
Abrasion resistance	97,3 %
Specific mass	645 g/l
Average pellet weight	5,3 g
Average pellet length	22,6 mm

Also available powdered on demand.

Product Presentation

*All SAFE® diets are available with different packaging, irradiation and with analytical data on demand.
Selected solutions of the most sold items.

DIET	STANDARD PACKAGING		USUALLY AVAILABLE WITH IRRADIATION DOSE
SAFE® A04	1 x 10 kg	Paper bag	
SAFE® A04 SP*	1 x 10 kg	Paper bag in plastic pouch	Min. 10 kGy, Min. 25 kGy
SAFE® R04*	1 x 10 kg	Paper bag, vacuum packed and boxed	Min. 10 kGy, Min. 25 kGy
SAFE® R04*	2 x 5 kg	Paper bag, double vacuum packed and boxed	Min. 25 kGy
SAFE® R04*	10 x 1 kg	Double vacuum packed and boxed	Min. 40 kGy
SAFE® A04C	1 x 10 kg	Double paper bag, certified	
SAFE® A04C SP*	1 x 10 kg	Double paper bag in plastic pouch, certified	Min. 10 kGy, Min. 25 kGy

Produced in France

PRODUCT DATA SHEET

Release date: December 2024

SAFE® A04

Ingredients

Barley, wheat, maize, soybean meal, wheat bran, hydrolyzed fish proteins, dicalcium phosphate, pre-mixture of minerals, calcium carbonate, pre-mixture of vitamins.

CENTESIMAL COMPOSITION

Cereals	84,1 %
Animal Proteins	4,0 %
Vegetal Proteins	8,0 %
Vitamins & Minerals	3,9 %

NUTRITIONAL COMPOSITION

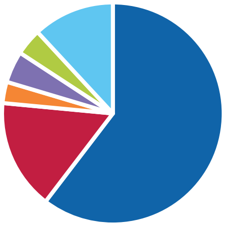

Nitrogen Free Extract	60,4 %
of which Starch	43,5 %
of which Sugars	3,2 %
Crude Protein	16,1 %
Crude Fat	3,1 %
Crude Ash	4,6 %
Crude Fiber	3,9 %
Moisture	11,9 %

ENERGY CONTENT

	MJ/kg	kcal/kg	%
ME Pig	13,2	3 145	
ME Atwater	14,0	3 339	
Energy from proteins	2,7	644	19,3
Energy from lipids	1,2	279	8,4
Energy from NFE	10,1	2 416	72,4

More information on energy calculation: www.safe-lab.com

For the welfare of animals SAFE® bedding and environmental enrichment such as SAFE® block gnawing logs and SAFE® nesting materials should be available in the cage.

Analysis End Product

TOTAL PER KG

AMINO ACIDS

Arginine	9 000 mg	Methionine	2 800 mg
Cystine	2 500 mg	Tryptophan	1 900 mg
Lysine	7 200 mg	Glycine	8 100 mg

FATTY ACIDS

Palmitic acid	5 900 mg
Stearic acid	600 mg
Palmitoleic acid	150 mg
Oleic acid	4 800 mg
LA	15 000 mg
ALA	1 200 mg

MINERALS

Calcium	7 300 mg
Phosphorus	5 500 mg
Sodium	2 500 mg
Potassium	6 000 mg
Magnesium	1 600 mg
Manganese	70,0 mg
Iron	270 mg
Copper	16,0 mg
Zinc	55,0 mg
Chlorine	4 000 mg

VITAMINS

Vitamin A	7 500 IU
Vitamin D3	1 000 IU
Vitamin E	30,0 IU
Vitamin K3	2,5 mg
Vitamin B1	5,0 mg
Vitamin B2	6,5 mg
Vitamin B3	70,0 mg
Vitamin B5	10,0 mg
Vitamin B6	3,0 mg
Vitamin B9	0,35 mg
Vitamin B12	0,010 mg
Biotin	0,080 mg
Choline	1 600 mg

The values of the end products are given as indication only and have no contractual value. They are calculated averages of product analysis results before irradiation and autoclaving. Depending on production conditions, storage and analytical methods variations may occur. An analysis is performed on request.

Produced in France

WORLDWIDE HEADQUARTERS
73494 Rosenberg (Germany)
sales@safe-lab.com

DIETS PRODUCTION SITE
89290 Augy (France)
info@safe-lab.com
