# Supplementary material for: Biombalance™, an Oligomeric Procyanidins-Enriched Grape Seed Extract, Prevents Inflammation and Microbiota Dysbiosis in a Mice Colitis Model
Source: Antioxidants (Basel). 2025 Mar 1;14(3):305. doi: 10.3390/antiox14030305 (PMC11939601; doi:10.3390/antiox14030305)
Supplement: Supplementary file 1 [file antioxidants-14-00305-s001.zip › TABLE S1.pdf]

**Category**	**Criterion**	**Definition**	**Score Range**
**Inflammation Severity**	Density and distribution of inflammatory cell infiltrates	0: No inflammation; 1: Minimal (small multifocal accumulation); 2: Moderate (small multifocal coalescing or big non-coalescing inflammation); 3: Severe (big multifocal and coalescing inflammation)	0–3
**Inflammation Extension**	Depth of inflammation through intestinal wall layers	0: No inflammation; 1: Mucosa; 2: Mucosa & submucosa; 3: Transmural	0–3
**Hyperplasia**	Crypt elongation relative to normal tissue	0: Normal; 1: <2-fold crypt height; 2: 2-fold crypt height; 3: 3-fold crypt height; 4: ≥4-fold crypt height, +/- adenomatous polyps	0–4
**Crypt Damage Severity**	Degree of crypt destruction and epithelial integrity	0: No crypt damage; 1: 1/3 of crypt damaged; 2: 2/3 of crypt damaged; 3: Crypts lost with intact surface epithelium; 4: Crypts lost with surface epithelium lost	0–4
**Crypt Damage Extension**	Percentage of affected area	0: No damage (0%); 1: Damage in 1–25%; 2: Damage in 26–50%; 3: Damage in 51–75%; 4: Damage in >76%	0–4

Total Histological Score

- The total score was calculated by summing the scores for *inflammation severity*, *inflammation extension*, *hyperplasia*, and *crypt damage severity*, then multiplying by the score for *crypt damage extension* (area involvement).
- Minimum possible score = **0**, Maximum possible score = **56** (if all criteria are included) or **40** (if crypt damage severity is excluded).

Protocol Details

- **Blinded Evaluation:** All slides were coded and scored double-blindly by trained pathologists.
- **Staining:** Colonic sections were stained with hematoxylin and eosin (H&E) for microscopic evaluation.
- **Assessment Methodology:** Scoring was performed under light microscopy at magnifications of ×100 and ×200.
- **Sample Size:** The study included *n=8 mice per group* for histopathological assessments, ensuring adequate statistical power.
