# Supplementary material for: Biombalance™, an Oligomeric Procyanidins-Enriched Grape Seed Extract, Prevents Inflammation and Microbiota Dysbiosis in a Mice Colitis Model
Source: Antioxidants (Basel). 2025 Mar 1;14(3):305. doi: 10.3390/antiox14030305 (PMC11939601; doi:10.3390/antiox14030305)
Supplement: Supplementary file 1 [file antioxidants-14-00305-s001.zip › Table S2.pdf]

Table xxSupp. RT-qPCR primer sequences.

Gene	Sequence 5'-3'	Annealing T °C	Accesion Number
Il-6	FW: TACCACTTCACAAGTCGGAGGC<br>RV: CTGCAAGTGCATCATCGTTGTTC	60	NM_031168
Il-17	FW: TTTAACTCCCTTGGCGCAAAA<br>RV: CTTTCCCTCCGCATTGACAC	61	NM_010552
Tnf-α	FW: GGTGCCTATGTCTCAGCCTCTT<br>RV: GCCATAGAACTGATGAGAGGGAG	60	NM_013693
Il-23	FW: AGCAACTTCACACCTCCCTAC<br>RV: ACTGCTGACTAGAACTCAGGC	62	NM_031252
Il-10	FW: AGAAGCATGGCCCAGAAATCA<br>RV: GGCCTTGTAGACACCTTGGT	62	NM_010548
Cxcl1	FW: GGGTGTTGTGCGAAAAGAAGTGC<br>RV: GGGAAGCGTCAACACGTGCG	62	NM_008176
Tgf-β	FW: CCGCAACAACGCCATCTATG<br>RV: CTCTGCACGGGACAGCAAT	62	NM_011577
Foxp3	FW: GGTACACCCAGGAAAGACAGC<br>RV: AAGACCTTCTCACAACCAGGC	62	NM_054039
F4/80 (Adgre1)	FW: CGTGTTGTTGGTGGCACTGTGA<br>RV: CCACATCAGTGTTCCAGGAGAC	60	NM_010130
iNOS	FW: GAGACAGGGAAGTCTGAAGCAC<br>RV: CCAGCAGTAGTTGCTCCTCTTC	60	NM_010927
Cat	FW: CGGCACATGAATGGCTATGGATC<br>RV: AAGCCTTCCTGCCTCTCCAACA	60	NM_009804
Sod1	FW: AACCAGTTGTGTTGTCAGGAC<br>RV: CCACCATGTTTCTTAGAGTGAGG	60	NM_011434
ZO-1	FW: GTTGGTACGGTGCCCTGAAAGA<br>RV: GCTGACAGGTAGGACAGACGAT	60	NM_009386.1
Ocln	FW: TGGCAAGCGATCATACCCAGAG<br>RV: CTGCCTGAAGTCATCCACACTC	60	NM_008756
Tlr4	FW: AGCTTCTCCAATTTTTCAGAACTTC	60	NM_021297

	RV: TGAGAGGTGGTGTAAGCCATGC		
Tlr5	FW: TCCTGACCAGAGCACATTTGCC<br>RV: CCTTCAGTGTCCCAAACAGTCG	60	NM_016928
Nod1	FW: GAAGGCACCCCATTGGGTT<br>RV: AATCTCTGCATCTTCGGCTGA	62	NM_172729
Nod2	FW: CCTAGCACTGATGCTGGAGAAG<br>RV: CGGTAGGTGATGCCATTGTTGG	60	NM_145857
Muc2	FW: ACCGCCTCACTCTGCCCCAA<br>RV: TGCAGCCACTGCCCGTGATG	62	NM_023566
Arg1	FW: CATTGGCTTGCGAGACGTAGAC<br>RV: GCTGAAGGTCTCTTCCATCACC	60	NM_007482
ChREBP	FW: CAGATGCGGGACATGTTTGA<br>RV: AATAAAGGTCGGATGAGGATGCT	62	NM_021455
Hprt	FW: GTTAAGCAGTACAGCCCCAAA<br>RV: AGGGCATATCCAACAACAAACTT	60-62	NM_013556

Thermal Cycling Program

1. **Initial denaturation and polymerase activation**: 95°C for 5 minutes
2. **Amplification** (40 cycles):
   - Denaturation: 95°C for 10 seconds
   - Annealing: 60°C for 30 seconds
   - Extension: 72°C for 30 seconds (with plate read)
3. **Final extension**: 72°C for 5 minutes
4. **Melt curve analysis**:
   - 65°C to 95°C with 0.5°C increments
   - 5 seconds per step
