# Supplementary figures and images for: Biombalance™, an Oligomeric Procyanidins-Enriched Grape Seed Extract, Prevents Inflammation and Microbiota Dysbiosis in a Mice Colitis Model
Source: Antioxidants (Basel). 2025 Mar 1;14(3):305. doi: 10.3390/antiox14030305 (PMC11939601; doi:10.3390/antiox14030305)

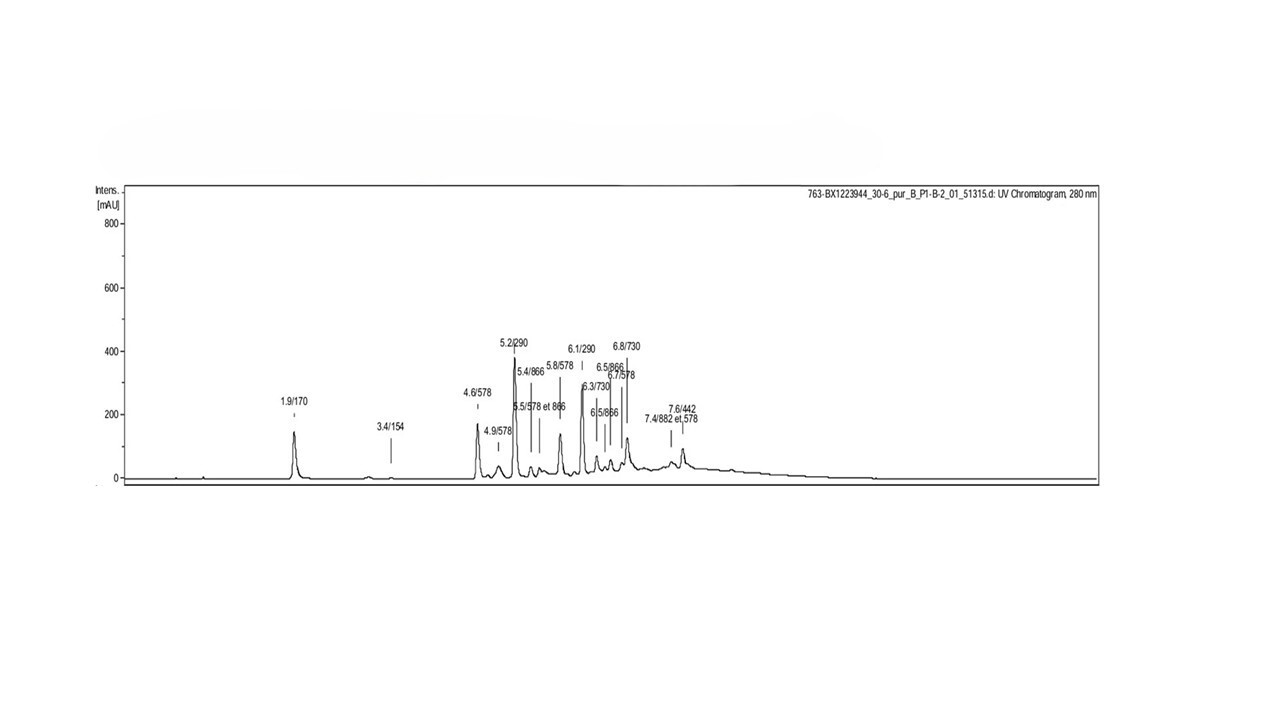

Supplement: Supplementary file 1 [file antioxidants-14-00305-s001.zip › S2 .JPG]

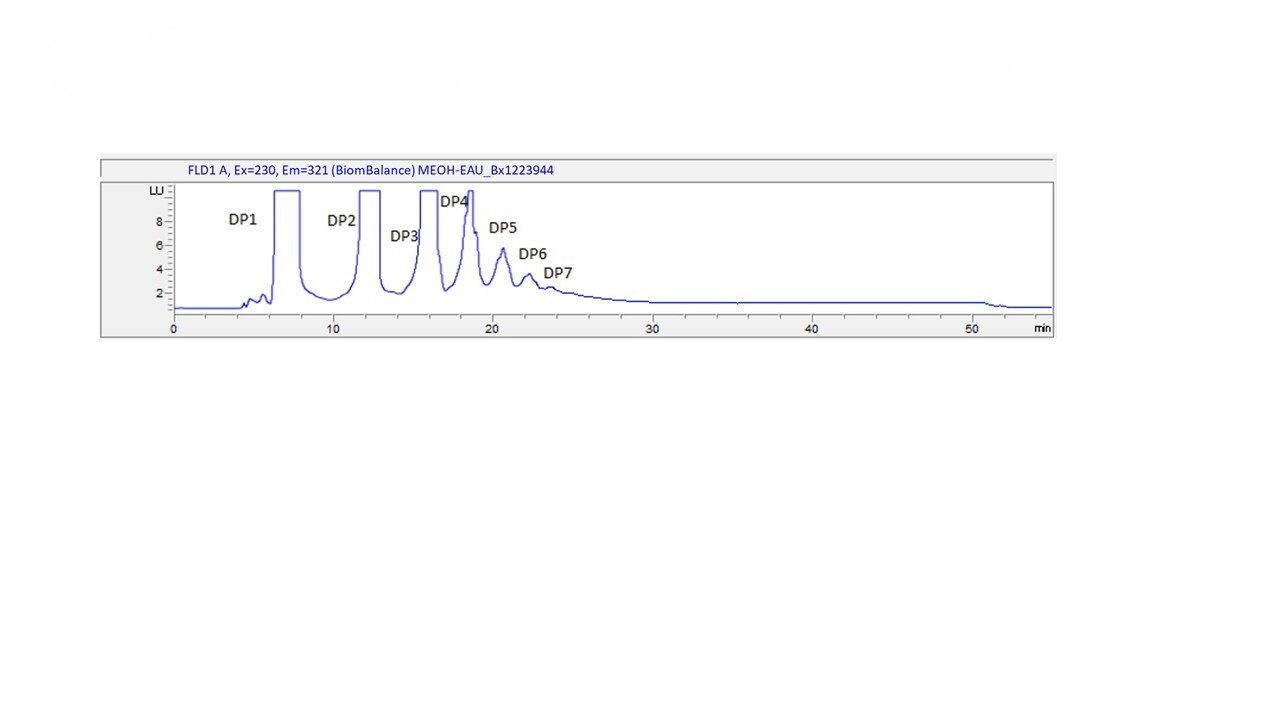

Supplement: Supplementary file 1 [file antioxidants-14-00305-s001.zip › S3.JPG]
